# Mountaintop Removal Mining: Digging Into Community Health Concerns

**DOI:** 10.1289/ehp.119-a476

**Published:** 2011-11-01

**Authors:** David C. Holzman

**Affiliations:** David C. Holzman writes on science, medicine, energy, economics, and cars from Lexington and Wellfleet, MA. His work has appeared in *Smithsonian*, *The Atlantic Monthly*, and the *Journal of the National Cancer Institute*.


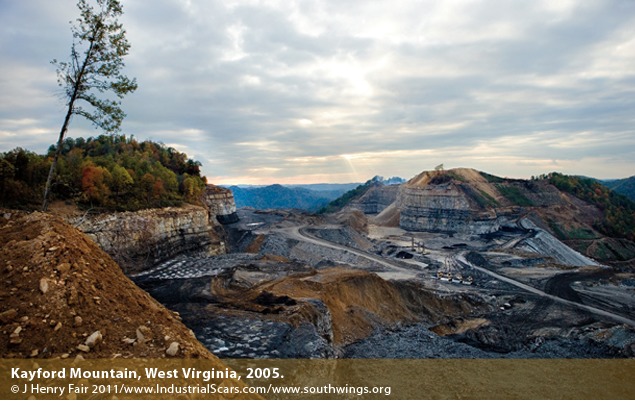


The practice of mountaintop removal (MTR) coal mining has been carried out on at least 500 Appalachian peaks.^1^ MTR mining is controversial for its environmental impacts: “Spoil”—the earth and rock dislodged by mining—is deposited in the valleys of this hilly and steep terrain,^2^ by some estimates burying almost 2,000 miles of headwater streams that ultimately feed the Mississippi River.^3^ Slurry, the residue from cleaning the coal, is impounded in ponds or injected into abandoned underground mine shafts, where it can leach potentially toxic constituents such as arsenic, lead, manganese, iron, sodium, strontium, and sulfate that ultimately may end up in groundwater.^4^ Now research studies are beginning to link these environmental impacts to adverse outcomes in community health, raising questions about whether the benefits of MTR mining come at too high a health cost.

For most of MTR mining’s history, permits had been relatively easy to obtain, but under the Obama administration, the U.S. Environmental Protection Agency (EPA) began conducting more stringent reviews of applications. By late January 2010, the agency had scrutinized roughly 175 proposed mines and signed off on only 48, according to *The Washington Post*.^5^ Then, on 1 April 2010, the EPA issued what it described as comprehensive guidance, based on strong science, designed to strengthen permitting requirements for Appalachian MTR and other surface coal mining projects.^6^

Subsequently, on 13 January 2011, in a decision that opponents of MTR mining considered a major victory, the agency halted disposal of mining waste at the proposed Spruce No. 1 Mine, which would have buried more than 6 miles of streams in Logan County, West Virginia, and dynamited roughly 3.5 square miles of mountaintop and forestland.^7^

**Figure:**
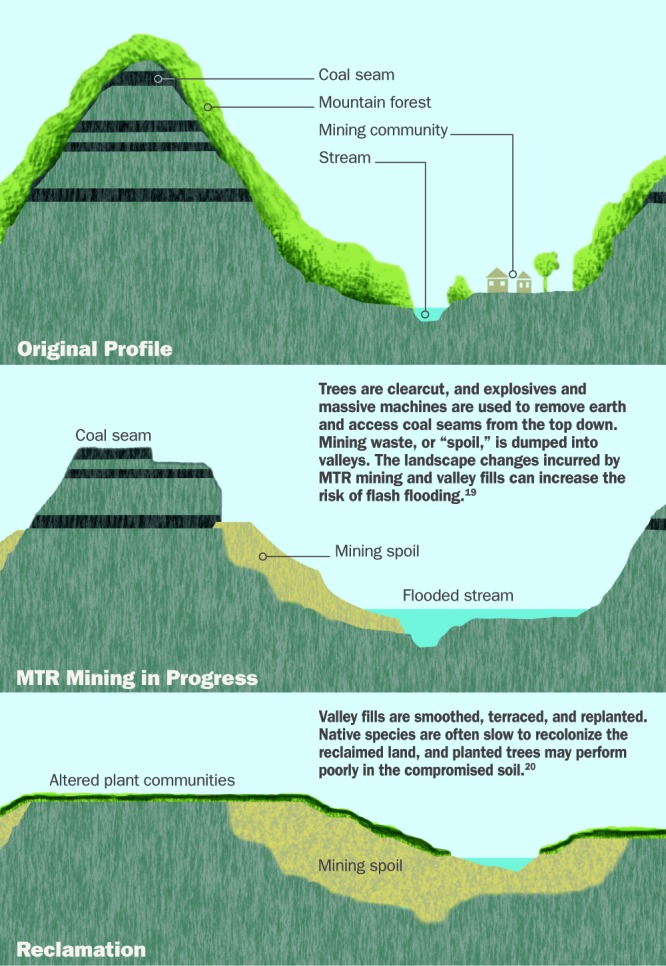
**MTR Mining in Progress.** Trees are clearcut, and explosives and massive machines are used to remove earth and access coal seams from the top down. Mining waste, or “spoil,” is dumped into valleys. The landscape changes incurred by MTR mining and valley fills can increase the risk of flash flooding.^19^ **Reclamation.** Valley fills are smoothed, terraced, and replanted. Native species are often slow to recolonize the reclaimed land, and planted trees may perform poorly in the compromised soil.^20^ Joseph Tart/EHP

A form of surface mining, MTR mining first emerged in the late 1960s but remained a small source of coal until the mid-1990s. Now it is a major form of coal mining in West Virginia and Kentucky—the second and third largest coal-producing states after Wyoming—and it also occurs in Virginia and Tennessee.^2^ A few factors account for its rise. First, the Clean Air Act amendments of 1990 encouraged companies to seek low-sulfur coal, which is abundant in central Appalachia. MTR mining also uses less labor than underground mining, with massive draglines able to move 100 cubic yards of earth in a single scoop. And with underground coal supplies significantly depleted, MTR mining allows the harvest of seams of coal too thin to work from traditional coal mines.^8^

The literature on health impacts of MTR mining has been both scant—encompassing a mere three-quarters of a page in the 99-page decision on the Spruce Mine^7^—and circumstantial. The dearth of literature is not surprising; just 10 investigators study the public health impact of MTR mining, says Michael Hendryx, an associate professor in the Community Medicine Department of West Virginia University in Morgantown. That’s partly because large-scale MTR mining is such a recent development. But a round of research articles published in 2011 has begun backing up anecdotal evidence of health effects with peer-reviewed data showing strong associations. The combination of the new studies along with the previous anecdotal and circumstantial evidence begs for more research to be conducted, Hendryx says.

## Pollution Pathways

One difficulty in studying potential health effects of MTR mining is that each coal formation has its own distinct chemistry. “Some have high selenium or high arsenic, and others don’t,” says Scott Simonton, a professor of environmental science at Marshall University in Huntington, West Virginia. Those elements, as well as manganese, lead, iron, and the compound hydrogen sulfide have been of particular concern regarding potential health effects, but “just about anything on the periodic chart can show up [in coal deposits],” Simonton says. (No studies have yet sought to identify organic compounds such as polyaromatic hydrocarbons. “We concentrate on inorganics because they’re easier to track,” Simonton notes.)

Pollutants may take any of several pathways into an area’s water supply. Some may leach into streams from the overburden that is dumped into valleys. Others hitch a ride in the slurry that is frequently injected directly into old mine shafts or impounded in ponds, from which it can seep through coal seams into ground-water. “Mining in general having an impact on groundwater is inarguable and well documented, even for surface mining,” Simonton says. “Geology and the impact that mining has on that geology guarantee that contaminated water will move out of the mine voids.” He adds, “I don’t think that the industry is even saying that the slurry doesn’t migrate out of the underground mines in which it is placed—they’re just saying it doesn’t hurt anyone.”

Where pollutants go once they hit groundwater is not easily predicted. Appalachian hydrology is complex and poorly charted, says Ben Stout, a professor of biology at West Virginia’s Wheeling Jesuit University. But severely contaminated water supplies have been the basis for multiple lawsuits against coal companies, alleging adverse health effects arising from contaminated drinking water. Residents may suddenly find that their water, which has been “great” for the previous 30–50 years, suddenly goes bad after mining begins nearby, Simonton says.

Dawn Seeburger, a toxicologist and owner of Environmental Resources & Consulting of Charleston, West Virginia, surveyed the parties to one of the lawsuits and read through their medical records for the plaintiffs’ attorney. She says the biggest health complaint was unremitting diarrhea. “Every family would complain about that, making statements that they could not leave home because they needed to have a restroom very close,” she says. Other conditions reported include learning disabilities, kidney stones, tooth loss, and some cancers.

**Figure:**
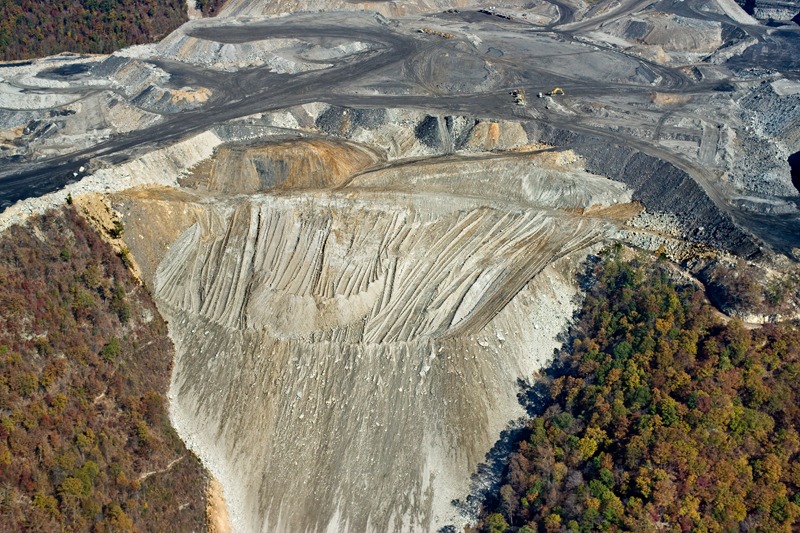
**Kayford Mountain, West Virginia** © J Henry Fair 2011/www.IndustrialScars.com/www.southwings.org

**Figure:**
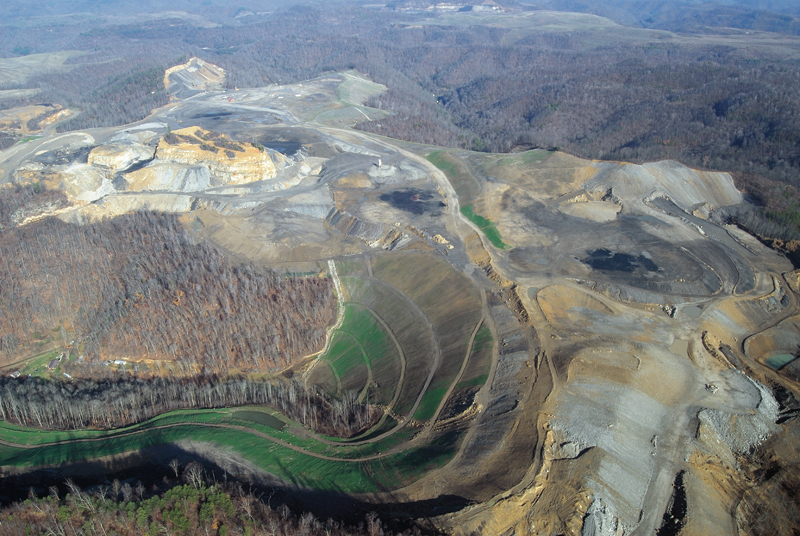
**Hazard, Kentucky.** Huge volumes of mining waste are dumped into valleys, covering an estimated 2,000 miles of streams to date and contributing to runoff high in potentially toxic metals and sulfur compounds. Companies are required by the Surface Mining Control and Reclamation Act to remediate valley fills to a quasi-natural state but may receive waivers from state agencies to leave the denuded land for commercial development. © 2011 George Wuerthner Photography

“Coal slurry has impacted at least one, and probably two communities in West Virginia where it was injected in such a way that the drinking water was contaminated,” says Carl Werntz, an associate professor in the Department of Community Medicine at West Virginia University. “People exposed to the water in their homes, both from consumption and showering, get what I call ‘slurry syndrome,’ a mixture of diarrhea, rash, some changes in their teeth, and increasing frequency of kidney stones,” he says. “Fortunately most of this went away when they got municipal water in the area.”

In Williamson, Merrimack, and Sprigg—three communities in Mingo County surrounding a site where coal slurry is injected into the ground—“some wells are in pretty good shape, and others are in horrible shape,” says Stout, who took samples from 18 area homes. “The first time I sampled, half the wells had lead above drinking water standards, a couple had [elevated] arsenic, and almost all violated iron and manganese standards.” Manganese concentrations were “way off the scale,” Stout says, ranging from nondetectable up to 4,063 ppb (the EPA recommends that manganese in drinking water not exceed 50 ppb^9^).

**Figure:**
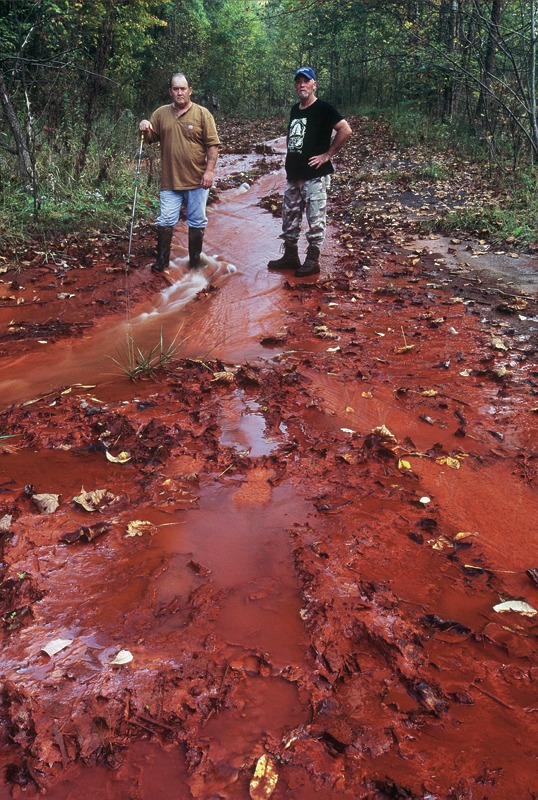
Metal-rich runoff pools below the mine site at Kayford Mountain, about 35 miles southeast of Charleston, West Virginia. Contaminated drinking water is one of the chief health concerns for communities surrounding MTR mining operations, although definitive links to reported health problems have not been established in these populations. © 2011 Mark Schmerling

It is impossible at this point to say definitively whether any of the adverse health conditions reported were caused by contaminated drinking water, but answers may be on the way: Kevin W. Thompson, a partner in Thompson Barney, PLLC, of Williamson, West Virginia, who brought one of the lawsuits on behalf of local residents, says the suit has already secured $5 million for a medical monitoring program. Thompson is soliciting proposals from area medical schools to conduct the program, and he hopes to award a contract by the end of the year.

Water contamination is not the only concern for communities. Simonton, who has surveyed “a couple of hundred homes,” mostly in Mingo and Boone counties, says that more than half smelled of hydrogen sulfide gas. He has measured concentrations in homes up to 21 ppm, compared with a tolerable concentration of 0.071 ppm for exposure durations of 1–14 days.^10^ Residents quickly become accustomed to the rotten-egg scent of hydrogen sulfide, but it is obvious to anyone who has not been chronically exposed, says Simonton, who explains the sulfide is produced when bacteria reduce sulfate that presumably comes from mining runoff.

“Sulfide has always been recognized as an occupational hazard,” Simonton says. However, the effects of long-term inhalational exposures like those in these homes have not been investigated, he says. Sulfide interferes with oxidative metabolism, and cardiac and nervous tissues are particularly sensitive, according to the World Health Organization.^10^ Chronic inhalational exposure in occupational settings has been shown to cause headache, irritability, and poor memory.^10^

Another potential hazard is coal dust from both mining and processing the coal. “The coal is crushed or pulverized, and that releases particulate matter into the air,” Hendryx says. In Sylvester, West Virginia, when a large processing plant was built in the town, “the air quality went through the basement,” he says. Local residents “had to wipe coal dust off of their furniture every day.”

Potential impacts from coal dust exposure include cardiovascular and lung disease, and possibly cancer. “That’s based on what we have observed in studies of general population outcomes for people that live in the mining areas,” Hendryx says. “This is also consistent with research by other investigators that has documented the environmental impacts of mining.”^11^ However, again, tests of health outcomes related to coal dust exposure have not been conducted in these populations.

**Figure:**
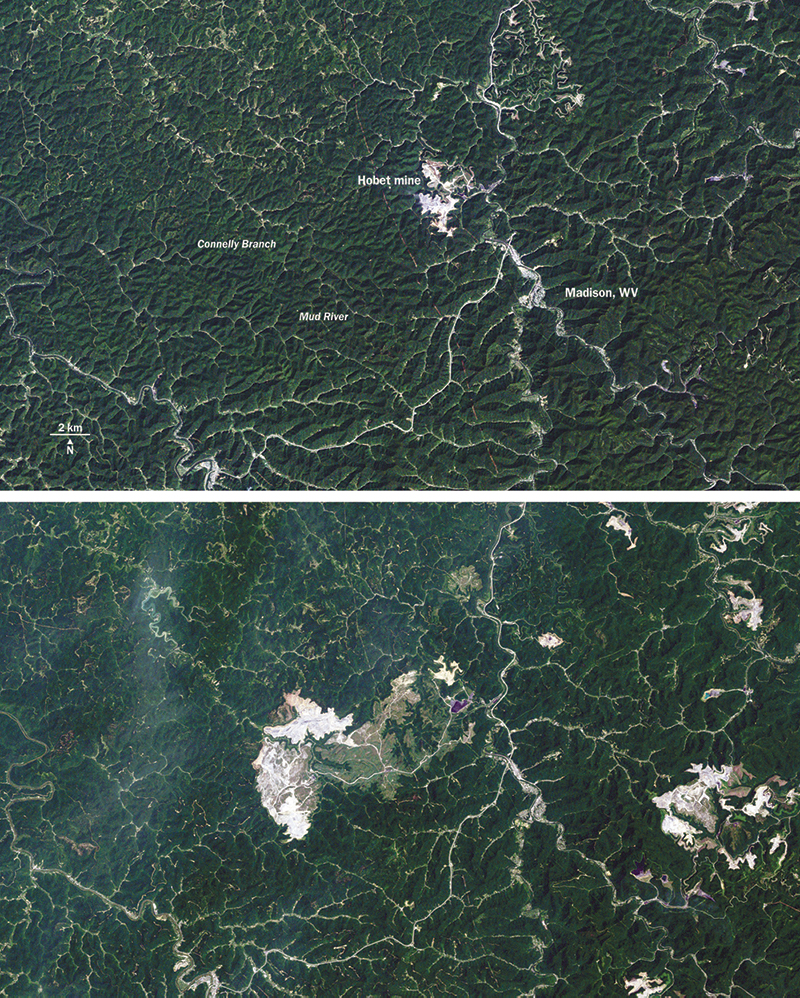
Images made from satellite data show the growth of West Virginia’s Hobet mine from 1984 (top) to 2009 (bottom). The natural landscape appears dark green, active mining areas appear off-white, and areas under reclamation appear light green. The Office of Surface Mining Reclamation and Enforcement is currently working on an environmental impact statement to support proposed revisions to increase environmental protections under the Surface Mining Control and Reclamation Act.^21^ Final revisions are expected in 2012. NASA

## Ecological Consequences,  Public Health

Health studies that have been conducted in Appalachia have revealed direct and indirect links to MTR mining. For starters, Gregory J. Pond, an environmental biologist with EPA Region 3 in Wheeling, showed that more than 90% of 27 Appalachian streams below valley fill sites were impaired as per Clean Water Act standards, while none of 10 streams sampled in nonmined valleys were impaired.^12^ The Clean Water Act specifies that streams must be suitable for “designated uses,” which include recreation, consumption of fish by humans, and protection of the health of aquatic life.^13^

To determine whether the streams were healthy, Pond and colleagues monitored benthic macroinvertebrates—insects and other invertebrates that are visible to the naked eye and live on the bottom of streams. These organisms respond predictably to stressors, and they “are the best indicators of stream health,” says Pond, noting that they have been used as such for more than a century. He adds that fish are often absent from small streams, reducing their utility as indicators.

“It turns out that in those places where the mining density is highest, the benthic communities are in the worst shape,” says Nathaniel P. Hitt, a research fish biologist in the Aquatic Ecology Branch of the U.S. Geological Survey’s Leetown Science Center in Kearneysville, West Virginia. “We’re not going to protect public health by restoring insects in streams,” he adds, but their disappearance is a warning for public health.

In a novel investigation, Hitt and Hendryx found that ecological impairment of streams correlated with human cancer mortality rates in surrounding areas. First they calculated a “stream condition index,” which reflects the presence of a healthy, well-functioning ecosystem. In this case they used metrics including the sum of taxonomic groups present, the sum of individuals from three specific taxa, and percentages from various other taxa. The cancers that rose with the declining stream condition index measure of impairment included respiratory, breast, and urinary cancers. Poverty, smoking, and urbanization, which predict cancer mortality, failed to account for the observed correlations.^14^

The investigators are quick to note that the correlations between stream quality impairment and the rates of certain cancers are only associations, not clearly cause and effect. Moreover, in the context of the study, mining activity is two steps removed from the cancers, and so any conclusion regarding causality would be a major stretch. However, Hitt and Hendryx wrote, such stream health assessments could contribute to a better understanding of human health: “Although [the West Virginia Department of Environmental Protection] conducts benthic macroinvertebrate sampling to assess the biological integrity of streams, our study reveals that these assessments may also improve our understanding of human health in nearby areas. As a result, biological monitoring and assessment may provide important social benefits.”

**Figure:**
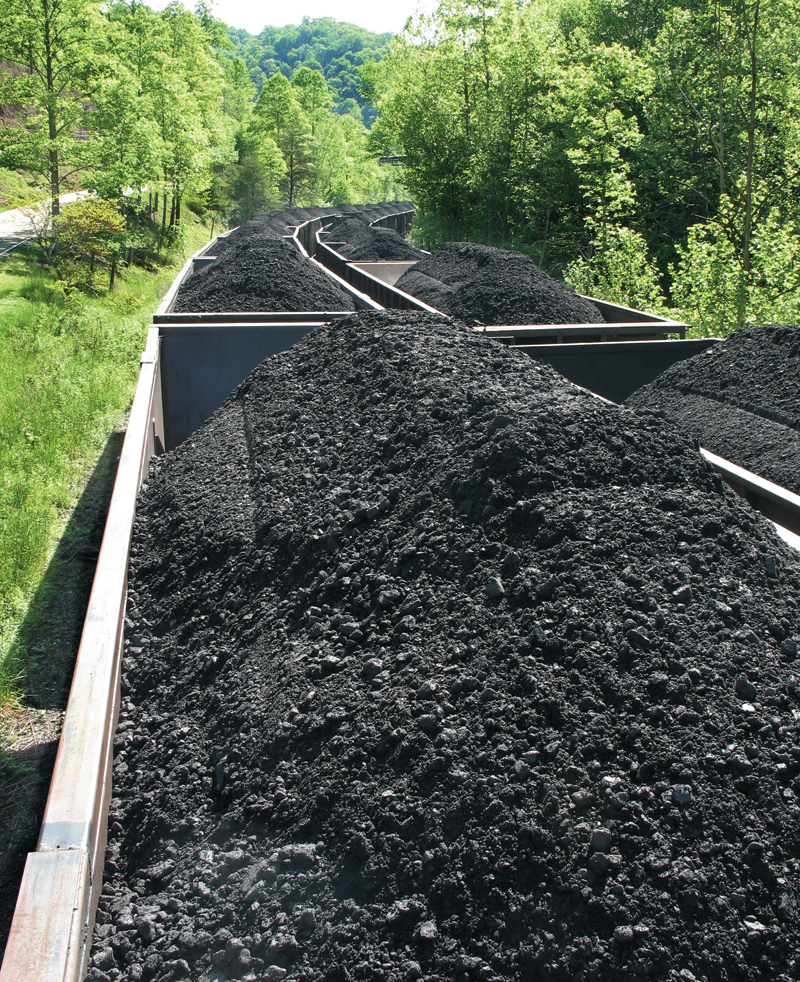
The Economics of MTR Mining Just how important is coal mining, including MTR mining, for the economy of Appalachia? According to the National Mining Association, MTR mining provides almost 60,000 direct and indirect jobs throughout Appalachia,^22^ which has a population of around 25 million.^23^ The average MTR mining wage earns a miner more than $66,000 per year, and MTR mining produces $5 billion of economic activity.^22^ In Kentucky, coal is a net drain on the state budget, according to a 2009 report by the Mountain Association for Community Economic Development, in Berea, Kentucky, with a net loss of more than $115 million for fiscal year 2006.^24^ That year, the state spent an estimated $643 million per year on infrastructure maintenance, environmental protection, research and development for the coal industry, educating public schoolchildren about coal, and state services for coal industry employees, and took in coal-related revenues of an estimated $528 million. Although coal represents only about 1% of employment in Kentucky, coal-mining counties are heavily dependent upon it—to their detriment, according to another report from the University of Kentucky. “This heavy dependence on the coal industry in Kentucky coal producing counties often leaves these counties susceptible to changes in the fortunes of the industry,” write the report authors. “As a result, losses in coal mining earnings in these counties often leads to increased poverty and dependence on social welfare programs. The Kentucky coal producing counties are also relatively more dependent on social welfare programs, including [Temporary Assistance for Needy Families] and Food Stamps, than other counties in the region.”^25^ © 2011 Christina Richards/Shutterstock

But other studies are making a more direct case for human health effects attributable to MTR mining. In 2011 three new studies showed strong associations between MTR mining and increased cardiovascular disease,^15^ increased frequency of birth defects,^16^ and reduced quality of life.^17^ In the first of these studies, Hendryx and colleague Laura Esch compared mortality data from MTR mining areas, conventional mining areas, and nonmining areas within the four Appalachian MTR mining states of Kentucky, Tennessee, Virginia, and West Virginia.^15^ They found 703 excess age-adjusted deaths from cardiovascular disease in MTR mining areas of the four states and 369 excess deaths in conventional mining areas, compared with nonmining areas within the same states. The study was limited in that Hendryx and Esch examined health effects at the county rather than individual level, making it impossible to determine the precise locations of health effects relative to MTR mines.

The second study, led by Melissa Ahern, a professor in the Department of Pharmaco-therapy at Washington State University in Spokane, found significantly higher rates of birth defects in MTR mining areas than in other mining and nonmining areas in the four MTR mining states.^16^ In this analysis of 1.9 million live births, children born in MTR mining counties were 26% more likely to have a birth defect than those born in nonmining areas, after adjusting for other risk factors such as maternal age, maternal alcohol consumption during preg-nancy, maternal diabetes, and low socioeconomic status. Prevalences of circulatory and respiratory system birth defects in MTR mining counties were nearly double those in other mining and nonmining counties. Furthermore, the overall prevalence of birth defects in MTR mining areas has been rising with the spread of MTR mining, with increases in the prevalence of several types of defects in the period 2000–2003 as compared with 1996–1999, but declines in others.

In the third study, Hendryx and Keith Zullig, an associate professor in the Department of Community Medicine at West Virginia University, obtained indivi-dual data through the Behavioral Risk Factor Surveillance System (BRFSS)—which has been gathering information on individuals throughout the United States since 1984—on health risk behaviors, preventive health practices, and access to health care, primarily related to chronic disease and injury.^17^ They calculated that people who lived in MTR mining areas had a 31% higher risk of reduced health-related quality of life (HRQOL)—or perceived physical and mental health over time—compared with people living in nonmining areas in the same states.

“Folks in mountaintop mining counties have 18 more unhealthy days per year than those in other [nonmining] counties,” Zullig says. Over a life span of 78 years, that adds up to nearly 4 additional years’ worth of impaired mental and/or physical health. The results suggest that previously documented HRQOL disparities in Appalachian coal-mining areas are concentrated in MTR mining areas. “These disparities partly reflect the chronic socioeconomic weaknesses inherent in coal-dependent economies and highlight the need for efforts at economic diversification in these areas,” wrote Hendryx and Zullig. “However, significant disparities persist after control for these risks and suggest that the environmental impacts of MTM may also play a role in the health problems of the area’s population.”^17^

## Slowing, but Not Stopping

Critics of MTR mining say changes in the EPA’s permitting practices are welcome but not sufficient. “The Obama administration has certainly done an improved job of scrutinizing individual projects,” says Jon Devine, an attorney with the Natural Resources Defense Council. These actions include addressing scientific issues that had previously been ignored and either stopping projects or forcing companies to make improvements. However, some projects are still allowed to proceed despite the fact there is no way to mitigate stream burial that likely will result.^7^

But in early October 2011, a U.S. district judge ruled that, during review of pending mine proposals, the EPA had overstepped its authority with the guidance it issued in April 2010, providing coordination and oversight to the U.S. Army Corps of Engineers and mine owners as to what is legal under the Clean Water Act.^18^ However, the ruling leaves the EPA with the option of vetoing mine permits, which it has previously been able to do. Devine says this probably means that more permits will be vetoed unless the Corps strictly follows the Clean Water Act.

Meanwhile, the evidence that MTR mining may directly and adversely affect public health has become significantly stronger since it was written in the Spruce Mine decision that the research studies to that point “by their nature could not and do not establish any causal linkage between surface coal mining and these elevated rates of adverse health effects.”^7^ Says Hendryx: “More research may still be needed, but the time has come to shift the burden of proof to the mining companies.”
